# The relationship between obstructive sleep apnea and atrial remodeling, fibrosis and inflammation

**DOI:** 10.1371/journal.pone.0328540

**Published:** 2025-08-20

**Authors:** Dasheng Lu, Kai Wang

**Affiliations:** 1 Department of Cardiology, The Second Affiliated Hospital of Wannan Medical College, Wuhu, Anhui Province, China; 2 Vascular Diseases Research Center of Wannan Medical College, Wuhu, Anhui Province, China; 3 Department of Geriatrics, The First Affiliated Hospital of Nanjing Medical University, Nanjing, Jiangsu Province, China; Tehran University of Medical Sciences, IRAN, ISLAMIC REPUBLIC OF

## Abstract

**Background:**

Obstructive sleep apnea (OSA), characterized by chronic intermittent hypoxia (CIH) is associated with atrial fibrillation. We explored the effects of OSA on atrial remodeling, fibrosis and inflammation, using both clinical and experimental studies.

**Methods:**

A total of 105 patients were grouped into control (n = 52), mild to moderate OSA (m-OSA, n = 27), and severe OSA (s-OSA, n = 26) groups. The participants underwent echocardiography and blood tests for interleukin-1β (IL-1β), tumor necrosis factor-α (TNF-α), collagen type 1 (col-1), and collagen type 3 (col-3). In order to eliminate the interference of obesity, an experimental study was performed using lean mice exposed to CIH.

**Results:**

In clinical patients, severe OSA featured higher body mass index (BMI) than control (28.12 ± 3.43 versus 24.40 ± 3.56 kg/m^2^, p < 0.001). Left atrial diameter was significantly higher in s-OSA group than in control group (38.35 ± 4.89 versus 35.39 ± 5.29 mm, P = 0.012). Patients with OSA had higher TNF-α levels than those in the control (P = 0.007). Consistently, plasma IL-1β levels were significantly elevated in the s-OSA and m-OSA groups compared with those in the control group. However, we did not observe significant changes in plasma col-1 and col-3 in patients with OSA. Subgroup analysis of patients with a BMI less than 25 kg/m^2^ showed that the inflammatory cytokine TNFα is correlated with OSA, rather than with BMI. In animal experiments, using lean mice exposed to IH for 3 weeks, WGA and Masson’s staining showed that IH enlarged the atrial myocardium and promoted atrial fibrosis. Atrial mRNA expression of TGFβ、col-1, col-3, IL-6, and TNF-α was significantly elevated after IH exposure.

**Conclusion:**

OSA, characterized by CIH, was associated with the increased atrial structural remodeling, fibrosis and inflammation.

## Introduction

Obstructive sleep apnea (OSA) syndrome is the most common form of sleep-disordered breathing, becoming a significant global health problem, with approximately 1 billion people in the general population suffering from OSA [1]. OSA appears to be strongly associated with the incidence of atrial fibrillation (AF). Among patients with AF, the prevalence of OSA ranged from 21% to 87% [2]. Moreover, OSA severity has been reported to be independently predictive of incidence of AF [3,4]. Chronic intermittent hypoxia (CIH) stands out as one of the key factors responsible for the atrial remodeling caused by OSA [5,6]. Previous studies have demonstrated that CIH by OSA contributed to left atrial electro-anatomical remodeling as well as alterations of atrial connexins [7,8], which ultimately promote the formation of an AF substrate.

Current evidence shows that treatment with OSA (CIH) may contribute to better outcomes in AF patients [9,10], some recent studies have suggested that continuous positive airway pressure (CPAP), an important therapy for OSA, did not prevent the incidence of AF [11] and the recurrence of AF after ablation [10]. It is possible that the current therapies for OSA do not have a well-targeted molecular basis and pathophysiology of AF. A better understanding of the mechanisms underlying the association between AF and OSA will help identify potential therapeutic strategies. Atrial remodeling and fibrosis are considered important factors in the formation of the substrate for AF [12]. We hypothesized that OSA and its primary pathological characteristic, CIH, are closely associated with left atrial enlargement, inflammation, and fibrosis.

## Materials and methods

The study was granted approval by the Institutional Review Board and the Ethics Committee of The Second Affiliated Hospital of Wannan Medical College, with the approval number WYEFYLS202106, on 2021-06-29. All procedures performed in this study involving human participants were in accordance with the ethical standards of the Second Affiliated Hospital of Wannan Medical College and with the 1964 Helsinki Declaration and its later amendments or comparable ethical standards.

The animal experiments were performed conform the NIH guidelines (Guide for the Care and Use of Laboratory Animals). All the procedures in this study were approved by the Ethics Committee of Wannan Medical College (LLSC-2021–189).

### Clinical participants

Adult patients with hypertension were enrolled from September 1, 2021 to December 31, 2021. Written informed consent was obtained from all the subjects. All patients were screened for snoring and scored using the NoSAS questionnaire. Patients with self-reported snoring or a NoSAS score of 8 or higher were selected to undergo polysomnography (PSG) to diagnose OSA. The apnea-hypopnea index (AHI) was calculated as the number of apnea and hypopnea episodes per hour of sleep. OSA was defined as AHI exceeding 5 [13]. According to the severity of OSA reflected by AHI, the participants were categorized into 3 groups: control group—patients without snoring and NoSAS score less than 8, plus those with recorded AHI less than 5/hour (52 patients); mild to moderate OSA-- AHI between 5/hour and 30/hour (27 patients), and severe OSA (AHI > 30/h; 26 patients). Subjects were excluded if they had a previous diagnosis of AF or prior antiarrhythmic therapy. Patients with inflammatory or autoimmune diseases or acute or chronic infectious disorders were excluded. We also excluded patients taking antibiotics.

Blood samples were obtained from all the enrolled patients under fasting conditions. Biochemical tests included measurement of blood lipid and fasting blood glucose levels. The remaining blood samples were centrifuged at 3000 rpm/4°C for 10 min, and the isolated plasma samples were collected and stored at −80°C until use. Plasma inflammatory cytokines, including interleukin-1β (IL-1β) and TNF-α, were analyzed using an Enzyme-Linked Immunosorbent Assay (ELISA) following the manufacturer’s instructions. Fibrosis-related indicators, including collagen type 1 (col1) and collagen type 3 (col3), were determined using commercial ELISA kits (Yubo, Shanghai, China).

Echocardiography was performed on the enrolled subjects using the Siemens SC2000 ultrasound system. Left atrial diameter (LAD), left ventricular diastole diameter (LVDd), interventricular septal thickness in diastole (IVSd), and ejection fraction (EF) were recorded. Blood pressure was measured using an electronic blood pressure monitor (Omron). The operators were blinded to patients’ disease.

***Patient and Public Involvement:*** Patients or the public WERE NOT were involved in the design, conduct, reporting, or dissemination plans of our research.

### Experimental studies

The animal study was approved by the Institutional Animal Care and Use Committee of Wannan Medical College in accordance with the NIH guidelines. Adult male C57/BL6J mice were randomly divided into two groups: control and IH. Mice were placed in a customized chamber and exposed to room air (control) or chronic intermittent hypoxia (IH, 6–7% O_2_ at nadir for 30 s followed by 21% O_2_, 30 cycles/h, by controlling the flow of N_2_). The IH mice were exposed to IH for 8 h per day for 3 weeks. At the end of the study period, all animals were euthanized by cervical dislocation. The heart samples were harvested for further analysis.

### Histological staining

Atrial tissues were stained with Masson’s trichrome for fibrosis. Wheat germ agglutinin (WGA staining was performed to determine cell size, as described prviously [14]. Imaging analyses were performed using Image-Pro Plus 6.0 software. At least five random microscopic fields were selected for quantitative analysis at X400 magnification.

### RNA extraction and quantitative real-time PCR

Total RNAs was extracted from atrial samples using TRIzol reagent (Ambio). cDNAs was synthesized using the HiScriptII Q RT SuperMix reverse transcription system (Vazyme, China). Quantitative real-time PCR (qRT-PCR) was performed using ChamQ SYBR qPCR Master Mix (Vazyme, China) according to the manufacturer’s instructions. The values were normalized to β-actin levels.

### Statistical analysis

Continuous data are expressed as the mean ± standard deviations (SDs) in Table. One-way ANOVA was used for comparison among multiple groups, followed by the LSD post hoc test for comparison between two groups. For the data for which the homogeneity of variance assumption was not met (Levene’s test), Dunnett’s 3T post hoc test was selected. For patients with specific AHI values and plasma parameters, we applied correlation analysis to evaluate the correlations between LAD and IL-1β, TNFα、col1 and col3 in plasma, BMI, and AHI. To normalize the distributions of the inflammatory cytokines IL-1β and TNFα, we applied logarithmic transformations to these variables. Categorical variables were shown as numbers and percentages, and between-group differences were determined using the χ2 test. Statistical analysis was performed using the SPSS version 16.0 software (SPSS, Chicago, IL, USA). Bar graphs were generated using the GraphPad Prism software. Statistical significance was set at P < 0.05.

## Results

### Clinical patient characteristics

A total of 105 patients were enrolled in this study. There were 52, 27, and 26 patients in the control, mild to moderate OSA (m-OSA), and severe OSA (s-OSA) groups, respectively. The participants’ characteristics and comparisons among the three groups are summarized in Table 1. There were no significant differences in sex, age, percentage of diabetes, fasting blood glucose, or lipid levels among the groups. However, patients with severe OSA featured higher BMI than patients in control (28.12 ± 3.43 versus 24.40 ± 3.56 kg/m^2^, p < 0.001). M-OSA patients also had higher value of BMI compared with control, but the difference did not reach statistical significant (26.01 ± 4.78 versus 24.40 ± 3.56 kg/m^2^, P = 0.081). The number of antihypertensive medications in the m-OSA and s-OSA groups was higher than that in the control group, but there was no significant difference in the current average blood pressure among the groups. There was no significant difference in the usage rate of statin drugs among the groups.

**Table 1:** Characteristics of patients with OSA and control participants.

Parameters	Control	m-OSA	s-OSA	P-value	95% confidence intervals^#^
**Gender (male, %)**	75.0	77.8	80.8	0.846	–
**Age (years)**	64.27 ± 11.62	53.44 ± 10.49	54.30 ± 10.58	0.944	–
**BMI (kg/m**^**2**^)	24.40 ± 3.56	26.02 ± 4.78	28.12 ± 3.43^*^	0.001	1.88 ~ 5.57
**Diabetes (%)**	19.2	18.5	26.9	0.698	–
**Mean SBP**	133.3 ± 11.5	137.4 ± 9.99	139.3 ± 13.1	0.072	–
**Number of antihypertensive medications**	1.51 ± 0.61	1.89 ± 0.75^*^	2.34 ± 0.80^*^	<0.001	0.50 ~ 1.17
**Echocardiographic Data**					
**LAD (mm)**	35.39 ± 5.29	35.90 ± 3.59	38.35 ± 4.79^*^	0.037	0.67 ~ 5.23
**LVDd (mm)**	46.12 ± 5.47	46.96 ± 4.16	48.42 ± 3.70	0.136	–
**IVSd (mm)**	8.69 ± 1.82	9.06 ± 1.60	9.24 ± 1.79	0.381	–
**EF (%)**	66.58 ± 5.65	65.52 ± 5.15	64.38 ± 4.39	0.684	–
**Biochemistry Analysis**					
**TC (mmol/L)**	4.40 ± 0.95	4.50 ± 0.79	4.68 ± 0.86	0.439	–
**TG (mmol/L)**	1.70 ± 1.32	1.96 ± 1.19	1.93 ± 0.79	0.554	–
**LDL-c(mmol/L)**	2.69 ± 0.74	2.75 ± 0.67	2.95 ± 0.72	0.311	–
**FBG(mmol/L)**	5.35 ± 1.38	5.29 ± 1.38	6.11 ± 1.56	0.061	–
**Statin use**	40.4%	51.9%	42.3%	0.612	–

Data are presented as mean ± standard deviation or percentages.

* Indicates significant difference versus control.

# The 95% confidence interval is only used when s-OSA is significantly different compared to the control, and it reflects the confidence interval of the effect size for the s-OSA group as compared to the control group.

OSA: obstructive sleep apnea; m-OSA: mild to moderate OSA; s-OSA: severe OSA; BMI: body mass index; AHI: apnea-hypopnea index; ODI: oxygen desaturation index; LAD: left atrial diameter; LVDd: left ventricular diameter in diastole; IVSd: interventricular septal thickness in diastole; EF: ejection fraction; TC: total cholesterol; TG: triglyceride; LDL-c: low density lipoprotein; FBG: fasting blood-glucose; SBP: systolic blood pressure.

### Clinical echocardiography parameters

Echocardiography tests showed that LAD was significantly higher in s- OSA group than in control group (38.35 ± 4.89 versus 35.39 ± 5.29 mm, P = 0.012), whereas no significant difference in LAD was observed between m-OSA and control (35.90 ± 3.59 versus 35.39 ± 5.29 mm, P = 0.655). There were no significant differences among the groups in LVDd, IVSd, and EF (P > 0.05) (Table 1).

### Patients inflammatory biomarkers and fibrosis related parameters

**Fig 1:**
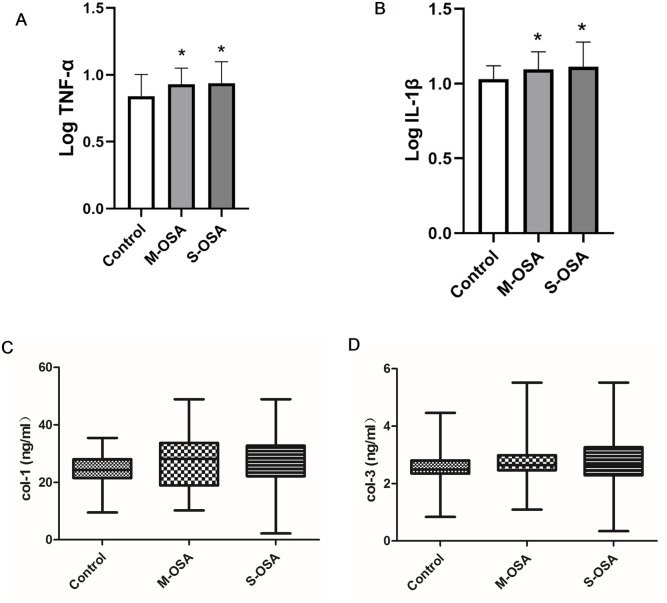
Plasma levels of inflammatory and fibrotic markers among mild to moderate OSA patients, severe OSA patients and controls. (A) shows that tumor necrosis factor alpha (log TNF-α) content was significantly increased in patients with mild to moderate OSA and severe OSA, and (B) shows that circulating interleukin 1β (log IL-1β) level elevated in both mild-to-moderate OSA (m-OSA) and severe OSA (s-OSA) patients. (C-D) show that circulating collagen type 1 (col-1) and (D) collagen type 3(col-3) were not significantly raised in OSA patients. The number of patients in the control, m-OSA, and s-OSA groups was 38, 22, and 20, respectively. Several patients were unwilling to undergo extra blood sampling except for routine biochemistry tests. Patients’ characteristics were similar to the total participants, namely, no significant differences in gender, age, percentage of hypertension and diabetes, fasting blood glucose and lipid level among groups were observed. *p < 0.05, versus the control.

We detected plasma levels of two significant inflammatory biomarkers (IL-1β and TNFα) as well as two main fibrosis-related biomarkers (col-1 and col-3). We observed significant differences in both TNF-αand IL-1βamong groups. Post-hoc comparisons showed that patients with s-OSA and m-OSA had higher TNF-α levels than controls (Fig 1A). Consistently, plasma IL-1β level was significant elevated in s-OSA and m-OSA group in comparison to control (Fig 1B). There was no statistically significant difference in plasma col-1 among groups (p = 0.37). Similarly, no significant changes were observed in plasma col-3 among groups (P = 0.58) (Fig 1C, 1D).

### Correlation between clinical parameters and plasma biomarkers

**Fig 2:**
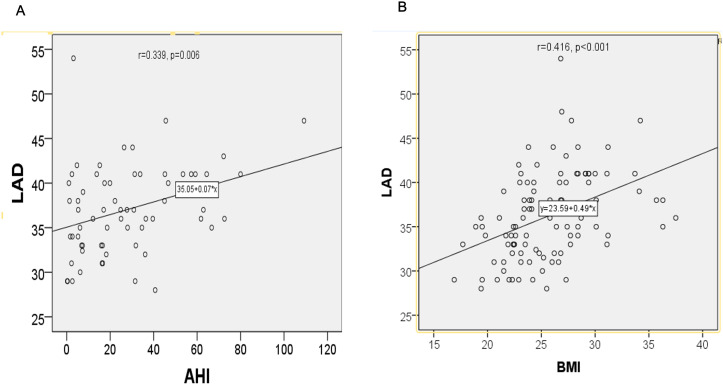
Correlations between LAD, inflammatory markers, AHI and BMI. Pearson correlation analysis shows that (A) left atrial diameter (LAD) significantly correlated with apnea-hypopnea index (AHI) and (B) body mass index (BMI) (n = 105). r indicates Pearson correlation coefficient.

LAD was positively correlated with AHI (r = 0.339, P = 0.006) (Fig 2A) and BMI (r = 0.416, P < 0.001) (Fig 2B). Moreover, there was a significant correlation between AHI and BMI (r = 0.314, P = 0.011). After adjusting for BMI, linear regression analysis showed that increased AHI remained associated with a larger LAD (P = 0.049). We observed similar results when AHI was replaced with the oxygen desaturation index (ODI), which is another parameter reflecting the severity of OSA.

We found no correlation between LAD and plasma levels of IL-1β, TNFα, col-1 and col-3 (all P > 0.05). Neither TNF-α nor IL-1β was significantly correlated with AHI, whereas the plasma level of TNF-α showed a positive correlation with BMI (rs = 0.321, P = 0.004).

### Subgroup analysis

Taking into account the differences in BMI between groups and its confounding effects, we conducted a subgroup analysis focusing on patients with a BMI < 25 kg/m^2^, a threshold chosen based on previous literature [15,16]. After stratification, there were no significant differences in BMI among the groups (p = 0.448). Our findings revealed that in these patients with a BMI < 25 kg/m^2^, the inflammatory cytokine TNFα was not significantly correlated with BMI (p = 0.128), but showed a Spearman positive correlation with the OSA group (p = 0.044), indicating that OSA was independently of BMI associated with inflammation.

### Animal experiments

#### Atrial remodeling following IH in lean mice.

**Fig 3:**
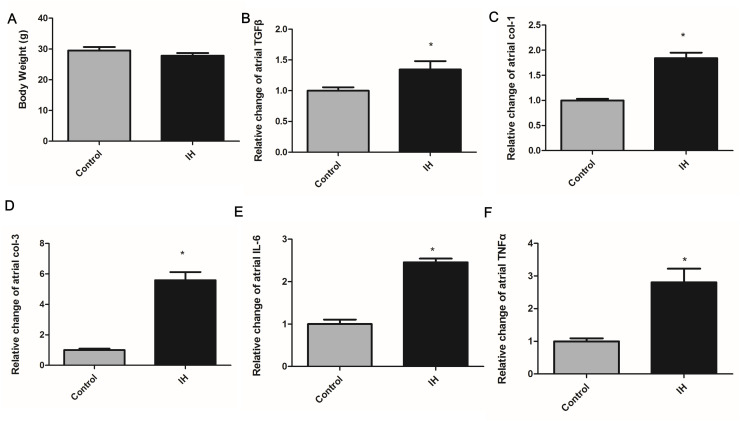
Changes of atrial mRNA expressions of inflammatory and fibrotic markers in lean mice exposed to intermittent hypoxia. (A) shows that body weight was similar between control and IH mice. Atrial mRNA expressions of fibrotic markers, including **(B)** TGFβ, (C) col-1 and (D) col-3, significantly increased in IH group. Atrial mRNA expressions of inflammation markers, including **(E)** IL-6 and **(F)** TNFα, were upregulated following IH. IH: intermittent hypoxia; TGFβ: transforming growth factor β1. *means P < 0.05 versus control (n = 6 per group).

**Fig 4:**
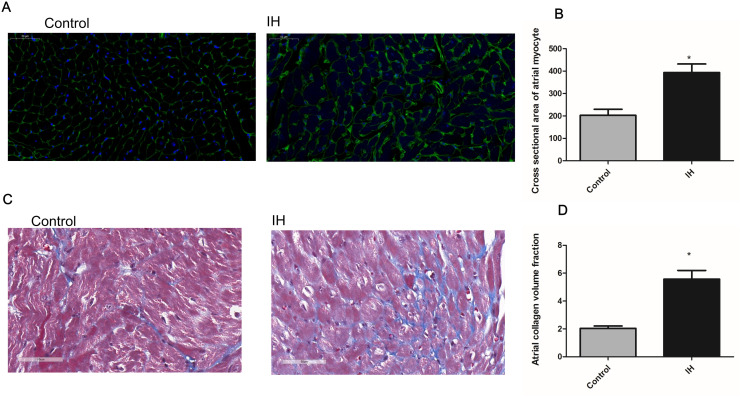
Atrial structural remodeling in mice with intermittent hypoxia. (A) Representative WGA staining images suggested atrial myocardium enlargement following IH; (B) statistical analysis showed that cross sectional of atrial myocardium was increased in IH group. (C) Representative Masson’s trichrome staining suggested that atrial fibrosis (blue area) was more evident in the IH group than in the control group; (D) Statistical analysis showed that atrial collagen volume fraction was higher in the IH group than in the control group. IH: intermittent hypoxia; CVF: collagen volume fraction. *indicates P < 0.05 versus control (n = 6 per group).

In lean mice exposed to IH for three weeks, no significant difference in body weight was observed (P = 0.26) (Fig 3A). We conducted WGA and Masson’s trichrome staining to assess atrial remodeling. Compared with the control group, IH enlarged the atrial myocardium and promoted atrial fibrosis (Fig 4). In contrast to the clinical data, the atrial mRNA expression of fibrosis markers, including TGFβ, col-1, and col-3, was significantly elevated after IH exposure (Fig 3B–3D).

#### Atrial inflammatory response after IH in mice.

We observed that the atrial mRNA expression of IL-6 and TNF-α was significantly higher in the IH group than in the control group (Fig 3E, 3F).

## Discussion

This study investigated the association of OSA and atrial structural remodeling, inflammation, and fibrosis, which are the major molecular mechanisms of AF. In clinical patients, we found that OSA was associated with increases in LA size and plasma levels of IL-1β and TNF-α. We also utilized a mouse model of CIH to mimic OSA, and animals with CIH exhibited remarkable atrial myocardium enlargement, inflammatory response, and collagen deposition.

The LA diameter is an easily accessible index for predicting the occurrence of AF in clinical practice. LA enlargement is associated with progression to chronic AF [17], and increases the risk of AF recurrence after a single circumferential pulmonary vein isolation [18]. In the present study, we found that LAD was significantly increased in patients with severe OSA, while patients with mild-to-moderate OSA had similar LAD values, indicating that OSA severity positively correlated to LA dilation, which was confirmed by correlation analysis. OSA-induced atrial remodeling holds significant clinical importance. Prior research has shown that OSA causes remodeling of the atrial structure by increasing atrial apoptosis, fibrosis, and alterations in the autonomic nervous system, which collectively contribute to the formation of a substrate that promotes the development of AF [19,20]. Our results indicate that reversing atrial remodeling could serve as a promising therapeutic approach for treating AF induced by OSA. In future research, it is advisable to incorporate specific outcomes related to AF to enhance the direct clinical relevance of the findings.

Besides LA dilation, tissue fibrosis is a hallmark of atrial structural remodeling and is considered a significant arrhythmogenic substrate in AF [21]. Atrial fibrosis is an important pathophysiological contributor and has been associated with AF recurrences [22]. Atrial fibrosis is mainly caused by increased collagen deposition, previous study by Duprez et al. suggested that the incidence of AF was strongly associated with circulating collagen markers, including plasma type I and type III collagen, in patients with no concurrent cardiovascular disease at baseline [23]. Little is known about the value of circulating collagen markers in OSA patients. Contrary to our expectations, the current study revealed that plasma levels of col-1 and col-3 in patients with OSA exhibited only a marginal increase, failing to achieve statistical significance. Notably, circulating collagen biomarkers are derived from collagen activity throughout the body, and OSA-induced atrial collagen deposition may not be sufficient to be distinguished in the circulation. Cardiac magnetic resonance imaging with late gadolinium enhancement or tissue‐specific assays would provide more robust evidence of fibrosis, which should be prioritized in future work. Inflammatory signaling pathways have been shown to create the substrate for AF development, and inflammatory biomarkers, such as TNF-α and IL-1β, were significantly associated with AF progression [24]. Existing evidence suggests that TNF-α and IL-1β lead to Ca^2+^ handling abnormalities in cardiomyocytes and subsequent arrhythmia [25]. In the current study, we found that OSA patients had elevated plasma IL-1β and TNF-α levels, and that patients with severe OSA had higher IL-1β concentrations than those with mild to moderate OSA, which may contribute to the onset of AF.

Although the clinical data in this study showed that OSA was associated with LA remodeling and inflammation, which may be responsible for the excess AF risk, it is difficult to identify whether OSA itself has a causal effect, due to the fact that OSA and AF share common risk factors and complications, including aging, obesity, and metabolic disorders. The groups did not exhibit clinically significant differences in terms of sex, age, or the morbidity of hypertension and diabetes. However, the OSA group had a higher BMI than the control group. Being overweight and obese are closely associated with inflammatory markers and are considered key risk factors for cardiovascular diseases [26]. Obesity is responsible for myocardial fibrosis through multiple pathways [27]. Additionally, obesity may contribute to AF substrates by altering inflammatory signaling pathways and cardiac structural remodeling [28]. In this study, we observed that BMI was positively correlated with OSA severity, LAD value, and circulating TNFα levels. It seems that BMI is an important confounding factor. However, after adjusting for BMI, AHI remained positively correlated with LAD. Furthermore, we conducted a subgroup analysis by stratifying patients based on BMI, and found that in these patients with a BMI < 25 kg/m2, the inflammatory cytokine TNFα was not significantly correlated with BMI, but showed a Spearman positive correlation with the OSA group, indicative of the independent role of OSA. In order to further eliminate the influence of obesity, we applied CIH model in non-obese mice. Numerous previous studies have employed the CIH model to investigate the effect of OSA on the heart [29,30]. We found that mice exposed to CIH exhibited atrial remodeling, including fibrosis, as reflected by WGA and Masson’s trichrome staining. In addition, atrial mRNA expression of fibrotic and inflammatory markers, including TGFβ, col-1, col3, IL-6, and TNFα, was upregulated following CIH exposure. These results indicated that CIH was associated with atrial remodeling, fibrosis, and inflammation. The findings align with the results of previous studies. Gami et al. demonstrated that OSA exerts an independent influence on AF beyond that of BMI [31]. Furthermore, Stafford et al. observed that the association between OSA and AF was observed exclusively in non-obese patients [32].

### Limitations

The present study had some limitations. Firstly, the sample size was relatively small, so the results should be interpreted with caution. Increasing the sample size in future studies would enhance statistical power and generalizability. Secondly, although the findings indicate that OSA directly contributes to an atrial substrate conducive to AF, potential residual confounding by obesity-related inflammation cannot be ruled out. In the real world, obese individuals are indeed more prevalent among patients with OSA, making it difficult to completely eliminate the confounding effect of obesity. However, this study has minimized the interference of BMI as a confounding factor by employing linear regression analysis, subgroup analysis and utilizing non-obese animal models. Thirdly, in the control group, only patients with snoring or a NoSAS score of no less than 8 underwent PSG to diagnose OSA. This risks misclassification—mild OSA can occur in low‐NoSAS individuals. However, the NoSAS questionnaire score (threshold of 8 points) had high discrimination power, and a low score presented a high predictive value for the exclusion of moderate/severe OSA [33,34]. Furthermore, the average BMI was 24.4 kg/m^2^ in the control group, and it has been reported that patients with mild snoring and a BMI lower than 26 are unlikely to have moderate or severe OSA [35]. Therefore, we can reasonably conclude that control patients were highly unlikely to have significant OSA. It’s important to mention that if there are some people with undiagnosed mild OSA in the control group, it would theoretically make the differences between the OSA group and the control group seem smaller. That means the results we found in our study should actually be even more significant. Fourthly, while CIH is a key pathological characteristic of OSA and has been widely adopted in animal models to mimic OSA, it’s important to clarify that CIH doesn’t fully equate to OSA. OSA can also exert influences on the atrial substrate through other mechanisms, including hypercarbia and intrathoracic pressure fluctuations [36]. OSA-related airway obstruction elevates intrathoracic pressure, mechanically stressing the atria and exacerbating chronic dilation and remodeling [37]. Hypercapnia significantly prolonged the effective refractory period and increased conduction time, suggesting its role in atrial electrical remodeling [38]. Future research must prioritize investigating the impact of these overlooked factors to advance our understanding of OSA-induced atrial remodeling. Future research must prioritize investigating the impact of these overlooked factors to advance our understanding of OSA-induced atrial remodeling. Besides, AF is caused by a complex combination of multiple factors, some of which are not discussed in this paper, including electrophysiological changes, connexin dysregulation, and Na + -channel dysfunction. Whether OSA promotes AF progression via these mechanisms remains unclear. Lastly, while the animal data demonstrate upregulation of TGF-β, IL-6, TNF-α, and collagen genes, but no exploration of upstream signaling. For example, NADPH oxidase 4 is closely associated with cardiac inflammation and fibrosis [39], and hypoxia-inducible factor 1 -regulated cytokines play a critical role in the pathogenesis of AF [40]. Future studies should elucidate these regulatory mechanisms to enhance mechanistic understanding.

## Conclusion

In summary, this study demonstrated that OSA was associated with the increased atrial structural remodeling, fibrosis and inflammation. These findings indicate that addressing inflammation and atrial remodeling resulting from OSA may offer potential targets for the prevention and treatment of OSA-associated AF. Future interventional studies should be conducted to assess whether treating OSA can attenuate atrial remodeling and lower the risk of developing AF.

## Abbreviations

AF: atrial fibrillation
AHI: apnea-hypopnea index
Col-1: collagen type 1
Col-3: collagen type 3
CPAP: continuous positive airway pressure
EF: ejection fraction
IH: intermittent hypoxia
IL-1β: interleukin-1β
IVSd: interventricular septal thickness in diastole
LAD: Left atrial diameter
LVDd: left ventricular diameter in diastole
OSA: obstructive sleep apnea
TNF-α: tumor necrosis factor α.
